# Medicinal Plants and Their Inhibitory Activities against Pancreatic Lipase: A Review

**DOI:** 10.1155/2015/973143

**Published:** 2015-11-10

**Authors:** Atefehalsadat Seyedan, Mohammed Abdullah Alshawsh, Mustafa Ahmed Alshagga, Sanaz Koosha, Zahurin Mohamed

**Affiliations:** ^1^Department of Pharmacology, Faculty of Medicine, University of Malaya, 50603 Kuala Lumpur, Malaysia; ^2^School of Biomedical Sciences, Faculty of Science, University of Nottingham Malaysia Campus, Jalan Broga, 43500 Semenyih, Selangor, Malaysia

## Abstract

Obesity is recognized as a major life style disorder especially in developing countries and it is prevailing at an alarming speed in new world countries due to fast food intake, industrialization, and reduction of physical activity. Furthermore, it is associated with a vast number of chronic diseases and disabilities. To date, relatively effective drugs, from either natural or synthetic sources, are generally associated with serious side effects, often leading to cessation of clinical trials or even withdrawal from the market. In order to find new compounds which are more effective or with less adverse effects compared to orlistat, the drug that has been approved for obesity, new compounds isolated from natural products are being identified and screened for antiobesity effects, in particular, for their pancreatic lipase inhibitory effect. Pancreatic lipase inhibitory activity has been extensively used for the determination of potential efficacy of natural products as antiobesity agents. In attempts to identify natural products for overcoming obesity, more researches have been focused on the identification of newer pancreatic lipase inhibitors with less unpleasant adverse effects. In this review, we consider the potential role of plants that have been investigated for their pancreatic lipase inhibitory activity.

## 1. Introduction

Obesity, which has been termed as the “New World Syndrome,” is now considered a global problem by the World Health Organization (WHO) and is associated with a vast number of chronic diseases and disabilities like dyslipidemia, fatty liver disease, osteoarthritis, hypertension, obstructive sleep apnea, gallstones, type 2 diabetes, reproductive and gastrointestinal cancers, coronary artery disease, heart failure, and stroke [1, 2]. Furthermore, it has also been recently claimed to promote breast cancer (in postmenopausal women) and also cancers of the endometrium, colon/rectum, pancreas, kidney, esophagus, gallbladder, liver, and prostate [3, 4].

Obesity is now recognized as the main life style disorder especially in developing countries and it is prevailing at an alarming speed in new world countries due to fast food intake, industrialization, and reduction of physical activity [5]. According to WHO, obesity kills more people than underweight and 65% of the population who live in developed countries are overweight [6]. It has been reported by the World Health Organization (WHO, 2014) that over 1.4 billion adults at the age of 20 and older were overweight, among whom almost 300 million women and more than 200 million men were obese [7]. In the United States, it has been reported that about one-third of the adult population is obese, and it has been considered a significant cause of human deaths [8]. In 2013, it was reported that in developing countries such as Malaysia about 44% of the adult men at the age of 20 and older were overweight and around 12% were obese. Rates are higher even among women, around 49% of the adult women at the age of 20 and older were overweight and around 17% were obese [9]. Obesity is considered an extremely costly health problem which in developed nations accounts for 2–6% of total health care costs [10].

Many medications have been used to prevent and manage obesity over the years. However, despite the seemingly unescapable progression of this disease and the promising results of some drugs on lowering of body weight and amendment of numerous cardiometabolic factors, in the last few years, most of the approved and marketed antiobesity drugs have been withdrawn from the market due to serious side effects [11]. In 2000, phentermine, an appetite-suppressant drug belonging to the family of *β*-phenethylamine, has been withdrawn by the European Medicines Agency (EMA), due to an undesirable risk to benefit ratio [11, 12]. Besides, mazindol and diethylpropion (amphetamine-like analogues) were withdrawn by the EMA in 2000 [12]. Rimonabant, an appetite-suppressant drug, is the first selective cannabinoid-1 (CB1) receptor blocker, and CB1 receptor plays a role in the regulation of appetitive behavior. In 2008, the use of rimonabant was suspended by EMA due to an increased risk of psychiatric side effects such as anxiety, suicidal ideation, sleep disorders, and depression. It is an appetite suppressant and was available in 56 countries from 2006 but it was never approved by the Food and Drug Administration (FDA) [13].

In 1997, sibutramine which is an anorectic or appetite suppressant, a selective noradrenaline/serotonin reuptake inhibitor, was used widely after approval by the FDA. In October 2010, FDA withdrew it from the market due to association with increased risk of serious nonfatal cardiovascular events like stroke and myocardial infarction [14]. Subsequently, in 2010 EMA also suggested suspending the use of sibutramine [14]. Common side effects of sibutramine are due to activation of the sympathetic nervous system such as insomnia, dry mouth, constipation, anorexia, headache, palpitation, and hypertension [15].

Currently only orlistat (Xenical), a drug which is considered to be acting through inhibition of pancreatic lipase enzyme, a key enzyme for the digestion of dietary triglycerides, has been approved by FDA and is available for long-term treatment of obesity. Orlistat is an inhibitor of gastrointestinal and pancreatic lipases which is able to prevent the absorption of approximately 30% of dietary fat [16]. Orlistat is the saturated derivative of lipstatin (isolated from a Gram-positive bacterium* Streptomyces toxytricini*) [17]. Apart from its antiobesity activity, it can also modestly decrease blood pressure, prohibit the onset of diabetes type 2, and improve oral glucose tolerance. In 1998, it was approved and is considered the only existing drug for the long-term control of obesity. The mechanism of lipase inhibition by orlistat is via covalent bonding to the lipase's active site serine [18].

Despite the promising results of orlistat for obesity treatment, it is associated with certain unpleasant gastrointestinal side effects [19]. These side effects result from orlistat's mode of action and the most important adverse effects are flatulence, liquid stools, diarrhea, oily spotting, incontinence or fecal urgency, and abdominal cramping [20]. Due to the adverse effects of orlistat, it may not be well tolerated. Hence, it is crucial to discover novel inhibitors, derived from natural sources particularly plants that are not associated with these serious side effects.

Several strategies have been applied for development of antiobesity agents including increase of energy expenditure (by blocking adipogenesis or inducing lipolysis followed by fat oxidation) and reduction of energy intake (by suppressing appetite and delaying or inhibiting absorption of nutrition). In this review, we consider the potential role of plants as antiobesity agents through investigation of their pancreatic lipase inhibition property.

## 2. Appetite Regulation

Appetite restriction is considered first line in obesity management [21]. Regulation of body weight via appetite control acts as a multifactorial action resulting from hormonal and neurological interrelationships. It is well known that dopamine, histamine, serotonin, and their related receptor activities are correlated with regulation of satiety [22].

A complex regulation of human appetite and satiety is made up of nearly 40 orexigenic and anorexigenic hormones, enzymes, neuropeptides, other cell signaling molecules, and their receptors [23]. The hunger and satiety signaling molecules are produced in the brain (centrally) and in liver, digestive tract, and adipose tissue (peripherally) [24]. The hypothalamus arcuate nucleus (ARC) is considered the most important area of the brain which plays a key role in appetite regulation. The appetite in the short term can be regulated by neural and endocrine signaling from gastrointestinal tract, while the information about adiposity level and acute nutritional status, from peripheral hormones, can be received and translated by the ARC and brainstem neurons [25].

Some substances like beta-adrenergic agonists are known to enhance hepatic fatty acid oxidation and decrease voluntary food intake in rats [26]. It is believed that, in the liver, energy status, mainly through production of ATP, activates signals via the vagal sensory neurons, to the appetite regulating centers of the brain [27]. Accordingly, once hepatic fatty acid oxidation is reduced, there is simultaneous reduction in ATP level which will then enhance appetite. Moreover, ingredients which enhance hepatic fatty acid oxidation like consumption of 1,3-diacylglyceride oil and medium-chain fatty acids lead to the reduction of food intake in human subjects [28].

The mechanism of appetite suppression in the brain is usually through affecting hunger control centers and is associated with a sense of fullness. However, reduction of food intake may increase ghrelin secretion in the stomach of animals and humans which leads to stimulation of increased intake. Thus, ghrelin antagonism might reduce or blunt the increased appetite which possibly happens with reduced feeding and, hence, might be considered an important target for treatment of obesity [29]. Besides, Melanin-Concentrating Hormone (MCH) receptor antagonism can also be considered a potential target for treatment of obesity via appetite regulation.

Moreover, fatty acid synthase (FAS) is the sole protein in the human genome, produced from acetyl coenzyme A and malonyl-CoA, known to catalyze the reductive synthesis of long chain fatty acids. Evidence indicates that inhibition of FAS in mice treated with FAS inhibitors leads to the reduction of food intake and hence reduction of body weight. Therefore, suppression of FAS can be a potential therapeutic target to inhibit appetite thereby inducing weight loss [30].

## 3. Inhibitors of Adipogenesis

Adipocytes, also known as fat cells and lipocytes, play a significant role in the control of energy balance and lipid homeostasis, through storing triglycerides and releasing free fatty acids in response to changing energy demands [31]. On the other hand, their long-term increased intake is associated with progression of obesity and leads to the serious comorbidities [32]. Therefore, adiposity mass and size are included as important markers of obesity. There are two types of obesity in which the first one is due to increase in adipocyte number (hyperplasic) and the second one is increase in adipocyte volume (hypertrophic). Hyperplasia is correlated more strongly with obesity severity and is most marked in severely obese individuals. However, hypertrophy, to a certain degree, is characteristic of all overweight and obese individuals [33]. Similar to adipose tissue and muscles, peripheral tissues deal with energy production and nutrient metabolism, whereas the central nervous system (CNS), specifically the hypothalamus, integrates and regulates energy expenditure and food intake [34]. Treatment which leads to the regulation of number and size of the adipocytes and the expression of signals related to energy balance and enhancement or inhibition of especial adipokines has been suggested to express antiobesity-related bioactivities. On the other hand, latest research findings indicated that inhibition of adipose tissue or adipogenesis expansion is associated with diabetes type 2 and other metabolic disorders, like atherosclerosis [35].

## 4. Inhibition of Fat Absorption

The digestion and absorption of nutrients should be decreased in order to reduce energy intake. As fat contributes more than protein or carbohydrate to unwanted calories deposition, inhibition of fat absorption can be considered the most common target to decrease energy intake. Among the existing treatments for obesity, development of nutrient digestion and absorption inhibitors is considered important strategies in the effort to decrease energy intake via gastrointestinal mechanisms [36]. Inhibition of digestion and absorption of dietary lipids, through an inhibitory action on pancreatic lipase, can be targeted for development of antiobesity agents [37].

Pancreatic lipase (triacylglycerol acylhydrolase), which catalyzes the digestion of dietary triglycerides, is an important lipolytic enzyme which is synthesized and secreted through the pancreas. In humans, pancreatic lipase (PL), encoded by the PNLIP gene, plays a significant role in dietary triacylglycerol absorption, hydrolyzing triacylglycerols to monoacylglycerols and fatty acids [38]. It is responsible for the hydrolysis of 50–70% of total dietary fats and is secreted into the duodenum via the duct system of the pancreas [36]. Pancreatic acinar cells secrete pancreatic lipase and this enzyme releases fatty acids from the triglyceride skeleton at the C-1 and C-3 position. These fatty acids are incorporated into bile acid-phospholipid micelles and further absorbed at the level of the brush border of the small intestine, to finally enter the peripheral circulation as chylomicrons. Interference with fat hydrolysis leads to decreased utilization of ingested lipids; hence, lipase inhibition reduces fat absorption.

## 5. Pancreatic Lipase Inhibitors from Natural Products

Pancreatic lipase inhibitory properties have been extensively examined for the determination of the potential effect of natural products as antiobesity agents. Due to the huge success of natural products for management of obesity, more research has been focused on the identification of newer pancreatic lipase inhibitors with less unpleasant adverse effects. So far, many natural products (plant extracts and isolated compounds) have been reported for their pancreatic lipase inhibition property including protamine [39], *ε*-polylysine [40], polysaccharides like chitosan [41], dietary fibers from wheat bran and cholestyramine [42], soya proteins [43], and synthetic compounds. The summary of medicinal plants as potential antiobesity agents with pancreatic lipase inhibitory activities reported in this review is presented in Table 1.

Sahib et al. [44] evaluated the* in vitro* antipancreatic lipase activity of ethanolic extract of* Centella asiatica*,* Morinda citrifolia*, and* Momordica charantia* (fruits) at various concentrations (7.81–250 ppm) using orlistat and epicatechin as synthetic and natural positive controls, respectively. The plant extracts* Morinda citrifolia*,* Momordica charantia,* and* Centella asiatica* fruits inhibited pancreatic lipase activity with 21.0 ± 1.3, 25.8 ± 0.1, and 25.3 ± 0.4% inhibition, respectively.

Bustanji et al. [45] screened the methanolic extracts of 23 traditional medicinal plants belonging to 15 families for their antipancreatic lipase activity regardless of their claimed ethnopharmacological uses. These plants were collected from several areas of Jordan. The inhibition of pancreatic lipase activity of plant extracts (12.5, 25, 50, and 200 *μ*g/mL) and orlistat (positive control) were measured using the spectrophotometric assay. Thirteen of the plant extracts inhibited the PL dose dependently, with an IC_50_ range between 108 and 938 *μ*g/mL, and these include* Anthemis palestina* Boiss. (107.7 *μ*g/mL),* Salvia spinosa* L. (156.2 *μ*g/mL),* Ononis natrix* L. (167 *μ*g/mL),* Fagonia arabica* L. (204.1 *μ*g/mL),* Origanum syriaca* (L.) (234 *μ*g/mL),* Hypericum triquetrifolium* Turra. (236.2 *μ*g/mL),* Malva nicaeensis* All. (260.7 *μ*g/mL),* Chrysanthemum coronarium* L. (286.1 *μ*g/mL),* Paronychia argentea* Lam. (342.7 *μ*g/mL),* Convolvulusalthaeoides* L. (664.5 *μ*g/mL),* Reseda alba* L. (738 *μ*g/mL), and* Adonis palaestina* Boiss. (937.5 *μ*g/mL). The positive control, orlistat, exhibited an IC_50_ value of 0.65 *μ*g/mL.

Ado et al. [46] evaluated the antilipase activity of the crude methanolic extracts of different parts (leaves, fruits, roots, seeds, stems, and flowers) of 98 plants from Malaysia, considered to be either herbal or aquatic plants. The obtained results showed that 19.4% of the extracts exhibited antilipase activity of more than 80%, while 22.4% showed moderate inhibition (41–80%) and 2% were neutral towards porcine pancreatic lipase (PPL) activity. The results indicated that the leaves of* Cynometra cauliflora* (*nam-nam*), the ripe fruit of* Averrhoa carambola*, leaves of* Aleurites moluccana* (L.) Willd. (candle nut/*buah keras*), and fruits of* Archidendron jiringa* (Jack) Nielsen L. showed the highest antilipase activity (100%) and are equivalent to 0.11 *μ*g orlistat/mL. Orlistat at 0.1 *μ*g/mL showed 95% inhibition of pancreatic lipase activity.

Adisakwattana et al. [47] evaluated the inhibitory activity of aqueous extract of different parts of 9 edible plants on pancreatic lipase* in vitro* using orlistat as positive control. The obtained result indicated that the* Ginkgo biloba* (ginkgo) and* Morus alba* (mulberry) exhibited promising inhibitory activities against pancreatic lipase. Among the plants, mulberry extract was considered the most effective pancreatic lipase inhibitor, whereas* Cassia angustifolia* (Senna) extract was considered the least potent inhibitor with IC_50_ of 0.01 ± 0.01 and 0.81 ± 0.03 mg/mL, respectively. On the other hand, all extracts were less potent inhibitors as compared to orlistat with IC_50_ of 1.34 ± 0.13 mg/mL.

Kim et al. [48] screened 115 herbal ethanol extracts for porcine pancreatic lipase inhibitory activity* in vitro*. Among the 115 plant species examined, eighteen extracts showed IC_50_ values of less than 50 *μ*g/mL, and three of these plants extracts, namely,* Solidago serotina* (whole plant),* Acer mono* (branches and leaves), and* Cudrania tricuspidata* (leaves), exhibited IC_50_ values of less than 10 *μ*g/mL. Remarkably,* Cudrania tricuspidata* showed an IC_50_ value of 9.91 *μ*g/mL. Then, the pancreatic lipase inhibitory effect of ethanol extract of* Cudrania tricuspidata* leaves (50 and 250 mg/kg) was investigated* in vivo*. The obtained results showed that* Cudrania tricuspidata* (50 mg/kg) decreased plasma triacylglycerol levels and the plant extract at the highest concentration (250 mg/kg) delayed lipid absorption significantly; however, these effects were weaker than that of orlistat (positive control).

Lai et al. [49] screened lipase inhibitory activity of methanolic extracts of different parts of 32 selected medicinal plants in Malaysia using porcine pancreatic lipase and p-nitrophenyl butyrate in an* in vitro* assay. Among the thirty-two extracts, twenty-seven crude extracts exhibited inhibitory activity against porcine pancreatic lipase* in vitro*.* Eleusine indica* exhibited the highest inhibitory effect against pancreatic lipase with 31.36 ± 0.58%, in comparison to the orlistat (34.49 ± 5.39%). Besides,* Myristica fragrans* (mace, 18–20%),* Melastoma candidum* 20%,* Phyla nodiflora* 18%, and* Dicranopteris linearis* 14% showed moderate activity (10–20%). On the other hand, nineteen crude extracts exhibited weak inhibitory activity (<10%) against PL.

Conforti et al. [50] screened pancreatic lipase inhibitory activity of 18 species of edible plants by monitoring the hydrolysis of p-nitrophenyl caprylate (p-NPC), which releases the yellow chromogen,* p*-nitrophenol. Among those examined, twelve extracts exhibited IC_50_ value more than 10 mg/mL:* Asparagus acutifolius*,* Silene vulgaris*,* Origanum vulgare*,* Raphanus raphanistrum*,* Smyrnium olusatrum*,* Sonchus asper*,* Foeniculum vulgare*,* Cichorium intybus*,* Papaver rhoeas,* and* Anchusa azurea*, while nine extracts exhibited IC_50_ value of less than 10 mg/mL. Orlistat at final concentration of 18 mg/mL was tested for comparison of inhibitory activity. The data indicated that, among plant extracts, those belonging to Lamiaceae (*Rosmarinus officinalis* and* Mentha spicata*) exhibited inhibitory activity with IC_50_ values of 7.00 mg/mL and 7.85 mg/mL, respectively. Among all plant extracts belonging to Asteraceae, only* Sonchus oleraceus* exhibited inhibitory activity (IC_50_ value of 9.75 mg/mL). The extract from* Diplotaxis tenuifolia*, belonging to Brassicaceae, exhibited inhibitory activity with IC_50_ value of 7.76 mg/mL. Besides, the aqueous ethanol extracts* Portulaca oleracea* (leaves) showed the highest inhibitory activity on lipase with IC_50_ value of 5.48 mg/mL.

Zheng et al. [51] screened lipase inhibitory activity of methanolic extracts of different parts of 37 traditional Chinese herbal medicines (0.2 mg/mL) against PPL* in vitro* using spectrophotometry with 2,4-dinitrophenyl butyrate. Among the 37 plant extracts examined, six extracts exhibited moderate to strong antilipase activity more than 30%.* Prunella vulgaris* L. (Labiatae) and* Rheum palmatum* L. (Polygonaceae) showed the highest inhibitory effect against PPL with 74.7% and 53.8%, respectively.* Polygonum cuspidatum* Sieb. et Zucc. (Polygonaceae),* Uncaria macrophylla* Wall. (Alismataceae),* Salvia miltiorrhiza* Bge. (Labiatae), and* Millettia reticulata* Benth. (Leguminosae) exhibited moderate activity with 30–40%. The result of inhibitory effects of* Rheum palmatum* L. and* Prunella vulgaris* L. at various concentrations revealed increasing inhibitory activities as concentration increased from 5 to 200 *μ*g/mL.

Toma et al. [52] investigated the inhibitory activity of the ethanolic extract of leaf of* Moringa stenopetala* on pancreatic lipase using spectrophotometric assay. The plant extract slightly inhibited pancreatic lipase with IC_50_ value of more than 5 mg/mL.

Teixeira et al. [53] studied the inhibitory activity of hydroethanolic extract of leaf of* Passiflora nitida* Kunth (1, 10, and 100 *μ*g/mL) on pancreatic lipase by a spectrophotometric assay using orlistat as positive control.* Passiflora nitida* extract at the highest concentration (100 *μ*g/mL) showed (67.6 ± 2.3%) pancreatic lipase inhibition and orlistat exhibited an inhibition of (74.0 ± 5.3%) at 1.6 *μ*g/mL. IC_50_ values for pancreatic lipase inhibition for* Passiflora nitida* extract and orlistat were 21.2 ± 0.8 and 0.1 ± 0.01 (*μ*g/mL), respectively.

Marrelli et al. [54] screened lipase inhibitory activity of hydroalcoholic extracts of five species of edible plants from Calabria region (Italy) against PPL* in vitro* using orlistat (20 *μ*g/mL) as control. Lipase activity was measured by monitoring the hydrolysis of p-NPC.* Clematis vitalba* L. and* Lepidium sativum* L. showed the highest inhibitory activity on pancreatic lipase with IC_50_ value of 0.99 ± 0.18 and 1.28 ± 0.29 mg/mL, respectively. Hence, the* Clematis vitalba* extracts can be considered a good candidate for more studies to isolate pancreatic lipase inhibitors.

Ekanem et al. [55] investigated the inhibition of pancreatic lipase of ethanolic extracts of *Aframomum melegueta *(seeds) and *Spilanthes acmella *(flower buds) at concentrations of 0.75–2.0 mg/mL using* in vitro* assay.* A. melegueta *and* S. acmella* (2 mg/mL) showed lipase inhibitory activities of 90% and 40%, respectively.

Kumar et al. [56] screened lipase inhibitory activity of different parts of 33 medicinal plants from India (n-hexane, dichloromethane, methanol, and ethyl acetate extracts)* in vitro*. Among the 33 plant extracts examined, the ethanolic extract of* Cassia siamea* roots (250 *μ*g/mL) exhibited the highest pancreatic lipase inhibition with 74.3 ± 1.4%. In addition, dichloromethane extract of* Lagerstroemia indica* fruits showed 61.2 ± 1.0% inhibition, and the ethyl acetate extract of* Chukrasia tabularis* leaves and bark showed 67.6 ± 2.1% and 63.7 ± 4.4% inhibition, respectively. Similarly, the ethanolic extract of* Vigna radiata* roots,* L. indica* fruits,* Justicia gendarussa* whole plant, and* Ferula asafoetida* resin showed 64.6 ± 0.2%, 70.1 ± 1.2%, 61.1 ± 2.6%, and 72.5 ± 3.5% PL inhibition, respectively. The methanol extract of* Ixora chinensis* flowers also showed 66.0 ± 2.1% enzyme inhibition.

The screening of the ethanolic extracts of 400 plants (100, 50, 25, 10, 5, 2.5, and 1.25 *μ*g/mL) using porcine pancreatic lipase assay* in vitro* led to the identification of several extracts with potential activity against PPL. Of the screened extracts, 44 extracts at the concentration of 100 *μ*g/mL exhibited high antilipase effect using 2,4-dinitrophenylbutyrate as a substrate in porcine pancreatic lipase assay. Among the extracts, four exhibited a relatively high antilipase activity of more than 30%.* Rubi Fructus* fruit,* Salicis Radicis* Cortex bark,* Geranium nepalense* whole grass, and* Cornus officinalis* fruit showed the significant inhibition of PPL with 32.5%, 34.8%, 38%, and 31.4%, respectively, compared to the orlistat (100 *μ*g/mL) as a positive control with 42% [57].

Kaewpiboon et al. [58] screened lipase inhibitory activity of 52 plant species (ethanol and water extracts) of Thai medicinal plants* in vitro*. The obtained data showed that, out of all the extracts, only ethanol extract of* Coscinium fenestratum* stems showed a weak lipase inhibitory activity (IC_50_ value of 160 *μ*g/mL) which had a 17.3-fold lower IC_50_ value than that of orlistat with 9.25 ± 1.25 *μ*g/mL.

Chompoo et al. [59] investigated the ability of acetone extract from flowers, seeds, leaves, pericarps, stems, and rhizomes of* Alpinia zerumbet* to inhibit pancreatic lipase* in vitro*. The obtained data showed that the seed extract (IC_50_ = 5.00 ± 0.07 *μ*g/mL) had the highest inhibitory effect on PL activity, amongst all different parts.

Morikawa et al. [60] investigated the ability of the methanol extract from bark of* Shorea roxburghii* to inhibit pancreatic lipase* in vitro*. The obtained result showed that the plant extract inhibited pancreatic lipase activity with IC_50_ of 31.6 *μ*g/mL compared to orlistat with IC_50_ of 0.056 *μ*g/mL.

Lee et al. [61] screened the* in vitro* lipase inhibitory effect of different parts of 61 medicinal plants (as ethanolic extract) from Korea by measuring the hydrolysis of p-nitrophenyl butyrate to p-nitrophenol. Among the 61 plant extracts examined,* Sorbus commixta* (leaf, stem) and* Viscum album* (whole plant) showed the best antilipase activity with IC_50_ values of 29.6 mg/mL and 33.3 mg/mL, respectively. Of the screened extracts,* Camellia japonica* (stem, leaf),* Castanea crenata* (staminate flower), and* Acer ginnala* (fruit) showed the inhibitory activity with IC_50_ value of 30–50 mg/mL. However, it was not more effective than positive control (orlistat) with IC_50_ value of 0.076 mg/mL.

Raghavendra et al. [62] studied the pancreatic lipase inhibitory activity of methanol extract of fruit pericarp* Artocarpus lakoocha* (10, 100, and 1000 mg/mL)* in vitro*. The plant extract inhibited pancreatic lipase in a dose dependent manner and highest inhibition (82.49%) was observed at concentration of 1000 mg/mL.

de Souza et al. [63] investigated the effect of the* Baccharis trimera* Less. leaf (ethanol, methanol, and aqueous extracts) on inhibition of pancreatic lipase activities* in vitro*. The aqueous and infused extracts did not exhibit inhibitory effect on the PL; however, the methanol extract considerably inhibited the PL activity by 78% and the ethanol extract presented low inhibition of 16% only.

Ivanov et al. [64] investigated the pancreatic lipase inhibitory activity of the crude aqueous ethanol extracts of* Bergenia crassifolia* rhizomes against human pancreatic lipase* in vitro* using fluorometric microplate reader. The plant extract inhibited human pancreatic lipase with IC_50_ value of 3.4 *μ*g/mL.

Yoshikawa et al. [65] investigated the effect of hot water-soluble extract from* Salacia reticulata* roots on inhibition of pancreatic lipase and lipoprotein lipase from adipose tissue using* in vivo* and* in vitro* assays. The plant extract inhibited pancreatic lipase and lipoprotein lipase (LPL) from adipose tissue with IC_50_ value of 264 mg/mL and 15 mg/mL, respectively. The plant extract inhibited LPL porcine and PL in rat adipocytes dose dependently.

Moreno et al. [66] assessed the pancreatic lipase and lipoprotein lipase activities of the* Vitis vinifera* L. (grape seeds) ethanol extract using* in vitro* assay. The plant extract inhibited pancreatic lipase activity dose dependently. At a concentration of 1 mg/mL, it exhibited the highest inhibitory effect against pancreatic lipase and lipoprotein lipase by 30% and 80%, respectively.

Moreno et al. [67] studied the inhibitory effects of ethanol extract of peanut (*Arachis hypogaea* L.) shells (hulls, seed coats) on lipoprotein lipase and human pancreatic lipase using* in vivo* and* in vitro* assays. The plant extract exhibited inhibitory activity on pancreatic lipase dose dependently (1 mg/mL = 42% inhibitory effect) and also exerted a mild inhibitory effect on lipoprotein lipase activity. Besides, the plant extract could prevent the body weight gain induced by feeding a high-fat diet to male Wistar rats for 12 weeks.

Adnyana et al. [68] assessed the effect of ethanol extract of pomegranate (*Punica granatum* L.) leaves with different concentrations on inhibition of pancreatic lipase using* in vitro* assay. The plant extract at concentration of 20.64 *μ*g/mL inhibited significantly the pancreatic lipase activity with IC_50_ of 50% compared to the orlistat as standard drug with various concentrations.

Habtemariam [69] investigated the inhibitory effects of ethanol extract of* Cassia auriculata* (aerial parts) on pancreatic lipase using* in vitro* assay. The plant extract inhibited pancreatic lipase in a dose dependent manner. It showed inhibitory activity against pancreatic lipase with IC_50_ value of 6.0 ± 1.0 mg/mL.

Han et al. [70] assessed the pancreatic lipase inhibitory activity of the water extract of* Juglans mandshurica* fruit using* in vitro* assay by measuring the rate of release of oleic acid from triolein. The plant extract strongly inhibited pancreatic lipase dose dependently. It inhibited pancreatic lipase activity with an IC_50_ value of 2.3 mg/mL.

Moreno et al. [71] investigated the inhibitory effect of ethanolic extract of mango tree (*Mangifera indica* L.) (leaves and stem bark) on pancreatic lipase, lipoprotein lipase, and hormone-sensitive lipase using* in vitro* assays. The plant extracts (stem bark and leaves) at a concentration of 1 mg/mL exhibited a significant inhibition of pancreatic lipase. The extract of stem bark (1 mg/mL) decreased LPL activity by 75%. The plant extract of both parts reduced the isoproterenol-stimulated lipolysis in 3T3-L1 adipocytes.

Ono et al. [72] assessed the effect of* Nelumbo nucifera* leaves (aqueous and ethanol extracts) on pancreatic lipase inhibition using* in vitro* and* in vivo* assays. The plant extracts inhibited lipase activity with IC_50_ value of 0.46 mg/mL and it promoted lipolysis in 3T3-L1 adipocytes. The obtained* in vivo* results showed that the plasma triacylglycerol level at 1 h after oral administration of a lipid emulsion to rats was elevated considerably and was reduced significantly in the group treated with the plant extract.

Kurihara et al. [73] investigated the inhibitory effect of* Cyclocarya paliurus* water extract (leaves) on pancreatic lipase activity. The plant extracts inhibited pancreatic lipase activity with IC_50_ value of 9.1 mg/mL. Besides, the extract (250 mg/kg) reduced plasma triacylglycerol levels in mice when fed with 5 mL/kg of lard and olive oil.

Kim and Kang [74] screened lipase inhibitory effect of the aqueous and ethanol extracts of different parts of 19 selected medicinal plants in Korea in an* in vitro* assay by a continuous-monitoring pH-Stat technique using tributyrin as a substrate. Among the nineteen plant extracts examined,* Illicium religiosum* (wood) and* Juniperus communis* (bark) showed the highest activity with an IC_50_ value of 21.9 and 20.4 *μ*g/mL, respectively, compared to the orlistat, with IC_50_ value of 0.750 *μ*g/mL. Meanwhile,* Thuja orientalis* (leaf),* Pyruspyrifolia* (bark and leaf), and* Euonymus alatus* (root) exhibited pancreatic lipase effect with IC_50_ value of 40–50 *μ*g/mL.

Sharma et al. [75] investigated antilipase activity of different parts of 75 medicinal plants belonging to different families using a radioactive method. Of all the tested extracts, methanolic extracts of whole part of three plants, namely,* Setaria italica* (L.) Palib.,* Orixa japonica* Thunb., and* Eriochloa villosa* (Thunb.) Kunth., showed strong* in vitro* antilipase activity (above 80%).

Gholamhoseinian et al. [76] investigated antilipase activity of methanol extract of 100 plants (various parts) using turbidimetric assay. Of the extracts tested,* Quercus infectoria* (galls),* Eucalyptus galbie* (leaves),* Rosa damascena* (flowers), and* Levisticum officinale* (roots) exhibited antilipase activity of more than 50%. On the other hand, the methanolic extracts of* Myrtus communis* (leaves),* Pistacia vera* (fruits hall),* Carthamus oxyacantha* (aerial parts),* Nigella sativa* (seeds),* Trigonella foenum-graecum* (seeds),* Urtica urens* (aerial parts),* Cinnamomum zeylanicum* (derm),* Rheum ribes* (rhizomes),* Pimpinella anisum* (seeds),* Ficus carica* (leaves),* Alhagi camelorum* (aerial parts),* Bunium persicum* (seeds),* Arctium lappa* (roots), and* Otostegia persica* (aerial parts) exhibited an inhibitory activity between 25 and 50% on pancreatic lipase.

Kwon et al. [77] investigated the inhibitory effect of* Dioscorea nipponica* methanol extract (roots) on pancreatic lipase activity by using 4-MU oleate as a substrate. The plant extracts inhibited lipase activity in a dose dependent manner. The extract at the concentration of 10 *μ*g/mL (IC_50_) inhibited pancreatic lipase significantly with 50% inhibition of enzyme activity compared to orlistat as standard drug with various concentrations.

## 6. Summary

Although there has been huge growth in the incidence of obesity over the last 25 years, progress in the discovery and development of new antiobesity drugs is rather limited. The only approved drug, orlistat, an inhibitor of gastrointestinal and pancreatic lipases, is associated with certain unpleasant gastrointestinal side effects. Hence, in search of nontoxic therapeutic, plant-based clinical products are considered an alternative option.

As drugs have failed to give desirable long-term results, it is significant to say that, in the last 10–20 years, a pervasive inspection began in order to clarify the most helpful source of new antiobesity compounds of natural products and to take over the present relative drug of doubtful effectiveness. In the last few years, interest in herbal medicines has increased and about 500 various plant species are used as key ingredients, while most are still being collected from the wild [78]. In recent years, treatments based on natural products and prevention of diverse pathologies have been the focal point and the vision has constantly spread globally. At the same time, proof and documentations have been gathered to support massive possibilities of medicinal plants which had been applied in various traditional systems [79]. For this reason, a focus on natural products among scientists is viewed as an appropriate alternative to multiple medications, and many are of the belief that natural products are potential source of new chemical substances with potential therapeutic efficacy, taking into account that traditional medicine is considered a significant source that may be utilized to control diverse diseases including obesity. Lately, different natural products and plants have been evaluated for their potential antiobesity effect both* in vivo* and* in vitro*. Today, the natural products seem to have the most interesting source of modern drugs and have revealed hopeful outcomes to combat obesity. Various studies identified new compounds and natural products for their PL inhibitory effect which are more potent compared to orlistat. Some of these plant extracts showed profound inhibition effects on fat digestion and are rich in polyphenols, saponins, and terpenes [37]. Many pancreatic lipase inhibitors from nature are under preclinical investigations; unfortunately, none of these have reached clinical level. In fact, it can sometimes be very challenging to extrapolate the results from* in vivo* or* in vitro* studies to human subjects, because they have not been found in many cases to be significantly effective. Therefore, the main limitation of previous studies is that most of the studied compounds have been shown to be more potent than orlistat but there have been no clinical studies to show their adverse effects as compared to orlistat.

In this paper, we have reviewed some natural products which have been investigated as a source of potential molecules with PL inhibitory activity. Hence, for all of them concerted efforts are required to define activities, mechanism of action, and optimal dose required as well as their possible toxic or side effects to nominate them as new antiobesity agents. Ideally, such research and exploration will lead to a more effective and safer pharmacological treatment of obesity.

Undoubtedly, there is a huge need for studying medicinal plants and their compounds as a therapeutic treatment to control obesity. Globally, there are huge numbers of unstudied medicinal plants, some of which have been used traditionally to control body weight, while only few of these ever reached the drug development stage due to the lack of scientific and clinical pharmacology data. Hence, it has not been possible for most of these to reach the stage of drug development in which it would be possible to determine the safety and efficacy of the herbal medicines. It is suggested that investigations should be increased to identify, isolate, and collect the compounds reported from plants so that their efficacy and pharmacological activities could be illuminated thoroughly, particularly those used as condiments and spices, in treating and overcoming obesity in humans. Improving knowledge on the use of antiobesity medicinal preparations is needed by continuously testing of the identified medicinal plants for bioactivity and toxicity, developing commercial formulations, and standardizing such extracts [80].

## Figures and Tables

**Table 1:** Summary of medicinal plants that showed inhibitory activity against pancreatic lipase.

Plant's name	Family	Plant part	Types of extract	Inhibitory activity against pancreatic lipase	Reference(s)
*Acer ginnala*	Aceraceae	Fruit	Ethanol extract	IC_50_ between 30 and 50 mg/mL	Lee et al. [61]

*Acer mono*	Aceraceae	Branches and leaves	Ethanol extract	IC_50_ less than 10 *μ*g/mL	Kim et al. [48]

*Adonis palaestina* Boiss.	Ranunculaceae	Aerial parts	Methanol extract	IC_50_ (937.5 *μ*g/mL)	Bustanji et al. [45]

*Aframomum melegueta*	Zingiberaceae	Seeds	Ethanol extract	90% inhibition	Ekanem et al. [55]

*Aleurites moluccana *(L.)	Euphorbiaceae	Leaves	Methanol extract	100% inhibition	Ado et al. [46]

*Alhagi camelorum*	Fabaceae	Aerial parts	Methanol extract	25–50% inhibition	Gholamhoseinian et al. [76]

*Alpinia zerumbet*	Zingiberaceae	Seeds	Acetone extract	IC_50_ (5 *μ*g/mL)	Chompoo et al. [59]

*Anchusa azurea*	Boraginaceae	Flowers	Aqueous ethanol	IC_50_ more than 10 mg/mL	Conforti et al. [50]

*Anthemis palestina *Reut. ex Boiss.	Asteraceae	Aerial parts	Methanol extract	IC_50_ (107.7 *μ*g/mL)	Bustanji et al. [45]

*Arachis hypogaea* L.	Fabaceae	Shells (hulls, seed coats)	Ethanol extract	42% inhibition	Moreno et al. [67]

*Archidendron jiringa *(Jack) I. C. Nielsen	Fabaceae	Fruits	Methanol extract	100% inhibition	Ado et al. [46]

*Arctium lappa*	Asteraceae	Roots	Methanol extract	25–50% inhibition	Gholamhoseinian et al. [76]

*Artocarpus lakoocha * Roxb.	Moraceae	Fruit	Methanol extract	82.49% inhibition	Raghavendra et al. [62]

*Asparagus acutifolius *L.	Asparagaceae	Stems	Aqueous ethanol	IC_50_ more than 10 mg/mL	Conforti et al. [50]

*Averrhoa carambola* L.	Oxalidaceae	Ripe fruit	Methanol extract	100% inhibition	Ado et al. [46]

*Baccharis trimera *Less.	Asteraceae	Leaves	Methanol and ethanol extracts	Methanol and ethanol extracts, respectively, showed 78% and 16% inhibition	de Souza et al. [63]

*Bergenia crassifolia*	Saxifragaceae	Rhizomes	Aqueous ethanol extracts	IC_50_ (3.4 *μ*g/mL)	Ivanov et al. [64]

*Bunium persicum*	Apiaceae	Seeds	Methanol extract	25–50% inhibition	Gholamhoseinian et al. [76]

*Camellia japonica* subsp. *rusticana*	Theaceae	Stem and leaves	Ethanol extract	IC_50_ between 30 and 50 mg/mL	Lee et al. [61]

*Carthamus oxyacantha*	Asteraceae	Aerial parts	Methanol extract	25–50% inhibition	Gholamhoseinian et al. [76]

*Cassia angustifolia * L.	Fabaceae	Leaves	Aqueous extract	IC_50_ (0.81 ± 0.03 mg/mL)	Adisakwattana et al. [47]

*Cassia auriculata* Linn.	Caesalpiniaceae	Aerial parts	Ethanol extract	IC_50_ (6.0 ± 1.0 mg/mL)	Habtemariam [69]

*Cassia siamea*	Caesalpiniaceae	Roots	Ethanol extract	74.3% inhibition	Kumar et al. [56]

*Castanea crenata*	Fagaceae	Staminate flower	Ethanol extract	IC_50_ between 30 and 50 mg/mL	Lee et al. [61]

*Centella asiatica*	Apiaceae	Fruits	Ethanol extract	25.3% inhibition	Sahib et al. [44]

*Chrysanthemum coronarium* L.	Asteraceae	Leaves and flowers	Methanol extract	IC_50_ (286.1 *μ*g/mL)	Bustanji et al. [45]

*Chukrasia tabularis *A. Juss.	Meliaceae	Leaves and bark	Ethyl acetate extract	Leaves and bark, respectively, showed 67.6% and 63.7% inhibition	Kumar et al. [56]

*Cichorium intybus* L.	Asteraceae	Leaves	Aqueous ethanol	IC_50_ more than 10 mg/mL	Conforti et al. [50]

*Cinnamomum zeylanicum*	Lauraceae	Derms	Methanol extract	25–50% inhibition	Gholamhoseinian et al. [76]

*Clematis vitalba* L.	Ranunculaceae	—	Hydroalcoholic extract	IC_50_ (0.99 *μ*g/mL)	Marrelli et al. [54]

*Convolvulus althaeoides* L.	Convolvulaceae	—	Methanol extract	IC_50_ (664.5 *μ*g/mL)	Bustanji et al. [45]

*Cornus officinalis*	Cornaceae	Fruits	Ethanol extract	31.4% inhibition	Roh and Jung [57]

*Coscinium fenestratum*	Menispermaceae	Stems	Ethanol extract	IC_50_ (160 *μ*g/mL)	Kaewpiboon et al. [58]

*Cudrania tricuspidata*	Moraceae	Leaves	Ethanol extract	IC_50_ (9.91 *μ*g/mL) *In vivo*: 50 mg/kg decreased plasma triacylglycerol levels and at 250 mg/kg delayed lipid absorption	Kim et al. [48]

*Cyclocarya paliurus*	Juglandaceae	Leaves	Aqueous extract	IC_50_ 9.1 mg/mL *In vivo*: 250 mg/kg reduced plasma triacylglycerol levels in mice	Kurihara et al. [73]

*Cynometra cauliflora * L.	Fabaceae	Leaves	Methanol extract	100% inhibition	Ado et al. [46]

*Dicranopteris linearis*	Gleicheniaceae	Aerial part	Methanol extract	14% inhibition	Lai et al. [49]

*Dioscorea nipponica*	Dioscoreaceae	Roots	Methanol extract	50% inhibition	Kwon et al. [77]

*Diplotaxis tenuifolia* L.	Brassicaceae	Leaves	Aqueous ethanol	IC_50_ (7.76 mg/mL)	Conforti et al. [50]

*Eleusine indica*	Poaceae	Aerial part	Methanol extract	31.36% inhibition	Lai et al. [49]

*Eriochloa villosa* (Thunb.) Kunth.	Poaceae	Whole plants	Methanol extract	Moe than 80% inhibition	Sharma et al. [75]

*Eucalyptus galbie*	Myrtaceae	Leaves	Methanol extract	More than 50% inhibition	Gholamhoseinian et al. [76]

*Euonymus alatus*	Celastraceae	Roots	Aqueous and ethanol extracts	IC_50_ between 40 and 50 *μ*g/mL	Kim and Kang [74]

*Fagonia arabica* L.	Zygophyllaceae	Aerial parts	Methanol extract	IC_50_ (204.1 *μ*g/mL)	Bustanji et al. [45]

*Ferula asafoetida*	Apiaceae	Resin	Ethanol extract	72.5% inhibition	Kumar et al. [56]

*Ficus carica*	Moraceae	Leaves	Methanol extract	25–50% inhibition	Gholamhoseinian et al. [76]

*Foeniculum vulgare* Miller subsp.	Apiaceae	Leaves and seeds	Aqueous ethanol	IC_50_ more than 10 mg/mL	Conforti et al. [50]

*Geranium nepalense*	Geraniaceae	Whole grass	Ethanol extract	38% inhibition	Roh and Jung [57]

*Ginkgo biloba* L.	Ginkgoaceae	Leaves	Aqueous extract	IC_50_ (0.05 ± 0.01 *μ*g/mL)	Adisakwattana et al. [47]

*Hypericum triquetrifolium* Turra.	Clusiaceae	Aerial parts	Methanol extract	IC_50_ (236.2 *μ*g/mL)	Bustanji et al. [45]

*Illicium religiosum *Sieb. et Zucc.	Schisandraceae	Woods	Aqueous and ethanol extracts	IC_50_ (21.9 *μ*g/mL)	Kim and Kang [74]

*Ixora chinensis* Lam.	Rubiaceae	Flowers	Methanol extract	66.0% inhibition	Kumar et al. [56]

*Juglans mandshurica *Maxim.	Juglandaceae	Fruits	Water extract	IC_50_ (2.3 mg/mL)	Han et al. [70]

*Juniperus communis*	Cupressaceae	Barks	Aqueous and ethanol extracts	IC_50_ (20.4 *μ*g/mL)	Kim and Kang [74]

*Justicia gendarussa *Burm. F.	Acanthaceae	Whole plant	Ethanol extract	61.1% inhibition	Kumar et al. [56]

*Lagerstroemia indica *(L.) Pers.	Lythraceae	Fruits	Dichloromethane extract	61.2% inhibition	Kumar et al. [56]

*Lepidium sativum* L.	Brassicaceae	—	Hydroalcoholic extracts	IC_50_ (1.28 *μ*g/mL)	Marrelli et al. [54]

*Levisticum officinale*	Apiaceae	Roots	Methanol extract	More than 50% inhibition	Gholamhoseinian et al. [76]

*Malva nicaeensis* All.	Malvaceae	Aerial parts	Methanol extract	IC_50_ (260.7 *μ*g/mL)	Bustanji et al. [45]

*Mangifera indica* L.	Anacardiaceae	Leaves and stem bark	Ethanol extract	75% inhibition	Moreno et al. [71]

*Melastoma candidum*	Melastomataceae	Aerial part	Methanol extract	20% inhibition	Lai et al. [49]

*Mentha spicata* L.	Lamiaceae	Leaves	Aqueous ethanol	IC_50_ (7.85 mg/mL)	Conforti et al. [50]

*Millettia reticulata* Benth.	Leguminosae	Rattan cane	Methanol extract	30–40% inhibition	Zheng et al. [51]

*Momordica charantia * L.	Cucurbitaceae	Fruits	Ethanol extract	25.8% inhibition	Sahib et al. [44]

*Morinda citrifolia* L.	Rubiaceae	Fruits	Ethanol extract	21% inhibition	Sahib et al. [44]

*Moringa stenopetala*	Moringaceae	Leaves	Ethanol extract	IC_50_ more than 5 mg/mL	Toma et al. [52]

*Morus alba *	Moraceae	Leaves	Aqueous extract	IC_50_ (0.01 ± 0.01 *μ*g/mL)	Adisakwattana et al. [47]

*Myristica fragrans*	Myristicaceae	Mace	Methanol extract	18–20% inhibition	Lai et al. [49]

*Myrtus communis*	Myrtaceae	Leaves	Methanol extract	25–50% inhibition	Gholamhoseinian et al. [76]

*Nelumbo nucifera* Gaertn.	Nymphaeaceae	Leaves	Aqueous and ethanol extracts	IC_50_ 0.46 mg/mL *In vivo*: it reduced plasma triacylglycerol level in rats	Ono et al. [72]

*Nigella sativa*	Ranunculaceae	Seeds	Methanol extract	25–50% inhibition	Gholamhoseinian et al. [76]

*Ononis natrix* L.	Fabaceae	Aerial parts	Methanol extract	IC_50_ (167 *μ*g/mL)	Bustanji et al. [45]

*Origanum syriaca* L.	Lamiaceae	—	Methanol extract	IC_50_ (234 *μ*g/mL)	Bustanji et al. [45]

*Origanum vulgare* L.	Lamiaceae	Stem and leaves	Aqueous ethanol	IC_50_ more than 10 mg/mL	Conforti et al. [50]

*Orixa japonica* Thunb.	Rutaceae	Whole plants	Methanol extract	More than 80% inhibition	Sharma et al. [75]

*Rosmarinus officinalis* L.	Lamiaceae	Leaves	Aqueous ethanol	IC_50_ (7.00 mg/mL)	Conforti et al. [50]

*Otostegia persica*	Lamiaceae	Aerial parts	Methanol extract	25–50% inhibition	Gholamhoseinian et al. [76]

*Papaver rhoeas* L.	Papaveraceae	Leaves	Aqueous ethanol	IC_50_ more than 10 mg/mL	Conforti et al. [50]

*Paronychia argentea* Lam.	Illecebraceae	Aerial parts	Methanol extract	IC_50_ (342.7 *μ*g/mL)	Bustanji et al. [45]

*Passiflora nitida* Kunth.	Passifloraceae	Leaves	Hydroethanolic extract	IC_50_ (21.2 *μ*g/mL)	Teixeira et al. [53]

*Phyla nodiflora* L.	Verbenaceae	Whole plant	Methanol extract	18% inhibition	Lai et al. [49]

*Pistacia vera * L.	Anacardiaceae	Fruits hall	Methanol extract	25–50% inhibition	Gholamhoseinian et al. [76]

*Pimpinella anisum*	Apiaceae	Seeds	Methanol extract	25–50% inhibition	Gholamhoseinian et al. [76]

*Polygonum cuspidatum *Sieb. et Zucc.	Polygonaceae	Root and rhizome	Methanol extract	30–40% inhibition	Zheng et al. [51]

*Portulaca oleracea* L.	Portulacaceae	Leaves	Aqueous ethanol	IC_50_ (5.48 mg/mL)	Conforti et al. [50]

*Prunella vulgaris* L.	Labiatae	Ear	Methanol extract	74.7% inhibition	Zheng et al. [51]

*Punica granatum* L.	Lythraceae	Leaves	Ethanol extract	50% inhibition	Adnyana et al. [68]

*Pyrus pyrifolia* (Burm.) Nak.	Rosaceae	Bark and leaf	Aqueous and ethanol extracts	IC_50_ between 40 and 50 *μ*g/mL	Kim and Kang [74]

*Quercus infectoria*	Fagaceae	Galls	Methanol extract	More than 50% inhibition	Gholamhoseinian et al. [76]

*Raphanus raphanistrum* L.	Brassicaceae	Leaves	Aqueous ethanol	IC_50_ more than 10 mg/mL	Conforti et al. [50]

*Reseda alba* L.	Resedaceae	Aerial parts	Methanol extract	IC_50_ (738 *μ*g/mL)	Bustanji et al. [45]

*Rheum palmatum* L.	Polygonaceae	Root and rhizome	Methanol extract	53.8% inhibition	Zheng et al. [51]

*Rheum ribes *	Polygonaceae	Rhizomes	Methanol extract	25–50% inhibition	Gholamhoseinian et al. [76]

*Rosa damascena*	Rosaceae	Flowers	Methanol extract	More than 50% inhibition	Gholamhoseinian et al. [76]

*Rubi Fructus*	Rosaceae	Fruits	Ethanol extract	32.5% inhibition	Roh and Jung [57]

*Salicis Radicis Cortex*	Ulmaceae	Bark	Ethanol extract	34.8% inhibition	Roh and Jung [57]

*Salacia reticulata*	Celastraceae	Roots	Hot water-soluble extract	IC_50_ 264 mg/mL	Yoshikawa et al. [65]

*Salvia miltiorrhiza* Bge.	Labiatae	Root and rhizome	Methanol extract	30–40% inhibition	Zheng et al. [51]

*Salvia spinosa* L.	Lamiaceae	Aerial parts	Ethanol extract	IC_50_ 156.2 *μ*g/mL	Bustanji et al. [45]

*Setaria italica* (L.) Palib.	Poaceae	Whole plant	Methanol extract	More than 80% inhibition	Sharma et al. [75]

*Shorea roxburghii*	Dipterocarpaceae	Bark	Methanol extract	IC_50_ (31.6 *μ*g/mL)	Morikawa et al. [60]

*Silene vulgaris* (Moench) Garcke	Caryophyllaceae	Leaves	Aqueous ethanol	IC_50_ more than 10 *μ*g/mL	Conforti et al. [50]

*Smyrnium olusatrum* L.	Apiaceae	Leaves	Aqueous ethanol	IC_50_ more than 10 *μ*g/mL	Conforti et al. [50]

*Sonchus oleraceus* L.	Asteraceae	Leaves	Aqueous ethanol	IC_50_ (9.75 mg/mL)	Conforti et al. [50]

*Solidago serotina*	Compositae	Whole plant	Ethanol extract	IC_50_ less than 10 *μ*g/mL	Kim et al. [48]

*Sonchus asper* (L.) Hill	Asteraceae	Leaves	Aqueous ethanol	IC_50_ more than 10 *μ*g/mL	Conforti et al. [50]

*Sorbus commixta* Hedl.	Rosaceae	Leaves and stem	Ethanol extract	IC_50_ (29.6 mg/mL)	Lee et al. [61]

*Spilanthes acmella*	Compositae	Flower buds	Ethanol extract	40% inhibition	Ekanem et al. [55]

*Thuja orientalis*	Cupressaceae	Leaves	Aqueous and ethanol extracts	IC_50_ between 40 and 50 *μ*g/mL	Kim and Kang [74]

*Trigonella foenum*-*graecum*	Fabaceae	Seeds	Methanol extract	25–50% inhibition	Gholamhoseinian et al. [76]

*Uncaria macrophylla* Wall.	Alismataceae	Aerial parts	Methanol extract	30–40% inhibition	Zheng et al. [51]

*Urtica urens*	Urticaceae	Aerial parts	Methanol extract	25–50% inhibition	Gholamhoseinian et al. [76]

*Vigna radiata*	Fabaceae	Roots	Ethanol extract	64.6% inhibition	Kumar et al. [56]

*Viscum album* L.	Loranthaceae	Whole plant	Ethanol extract	IC_50_ (33.3 mg/mL)	Lee et al. [61]

*Vitis vinifera* L.	Vitaceae	Seeds	Ethanol extract	30% inhibition	Moreno et al. [66]

IC_50_: inhibition concentration 50.
